# The Shinyanga Patient: A Patient’s Journey through HIV Treatment Cascade in Rural Tanzania

**DOI:** 10.3390/ijerph18168418

**Published:** 2021-08-09

**Authors:** Nwanneka E Okere, Veryeh Sambu, Yudas Ndungile, Eric van Praag, Sabine Hermans, Denise Naniche, Tobias F Rinke de Wit, Werner Maokola, Gabriela B Gomez

**Affiliations:** 1 Department of Global Health, Amsterdam UMC, University of Amsterdam, 1105 AZ Amsterdam, The Netherlands; s.hermans@aighd.org (S.H.); t.rinkedewit@aighd.org (T.F.R.d.W.); 2 Amsterdam Institute for Global Health and Development, 1105 BP Amsterdam, The Netherlands; eric.vanpraag@gmail.com; 3 National AIDS Control Programme, Dodoma 41110, Tanzania; veryeh77@hotmail.com (V.S.); drwernerm@yahoo.com (W.M.); 4 Regional Health Management Team, Shinyanga 37103, Tanzania; yudas.ndungile@gmail.com; 5 ISGlobal-Barcelona Institute for Global Health, Hospital Clinic, University of Barcelona, 08036 Barcelona, Spain; denise.naniche@isglobal.org; 6 Department of Global Health and Development, London School of Hygiene and Tropical Medicine, London WC1E 7HT, UK; gabriela.gomez@lshtm.ac.uk

**Keywords:** patient-pathway analysis, HIV, sub-Saharan Africa, health services, access

## Abstract

The 2016–2017 Tanzania HIV Impact Survey (THIS) reported the accomplishments towards the 90-90-90 global HIV targets at 61-94-87, affirming the need to focus on the first 90 (i.e., getting 90% of people living with HIV (PLHIV) tested). We conducted a patient-pathway analysis to understand the gap observed, by assessing the alignment between where PLHIV seek healthcare and where HIV services are available in the Shinyanga region, Tanzania. We used existing and publicly available data from the National AIDS Control program, national surveys, registries, and relevant national reports. Region-wide, the majority (*n* = 458/722, 64%) of THIS respondents accessed their last HIV test at public sector facilities. There were 65.9%, 45.1%, and 74.1% who could also access antiretroviral therapy (ART), CD4 testing, and HIV viral load testing at the location of their last HIV test, respectively. In 2019, the viral suppression rate estimated among PLHIV on ART in the Shinyanga region was 91.5%. PLHIV access HIV testing mostly in public health facilities; our research shows that synergies can be achieved to improve access to services further down the cascade in this sector. Furthermore, effective engagement with the private sector (not-for-profit and for-profit) will help to achieve the last mile toward ending the HIV epidemic.

## 1. Introduction

The goal of the Joint United Nations Program on HIV/AIDS (UNAIDS) strategy is that all persons living with HIV (PLHIV) have equitable access to diagnosis and treatment [1]. Anchored on three key stages in the HIV treatment cascade, the 90 – 90 – 90 targets by 2020 of this strategy have presented simple benchmarks for measuring the effectiveness of HIV programs. These targets (90% of all PLHIV knowing their HIV status, 90% of people who know their HIV-positive status having access to treatment, and 90% of people on treatment having suppressed viral load) have provided a uniform system of reporting and comparing performance between countries. By the end of 2019, global estimates for progress towards the targets stood at 81-82-88, with wide variability in achievement between countries, and the lowest achievement recorded in low- and middle-income countries (LMICs) [2]. The targets proposed a stretch to 95 – 95 – 95 by 2030 if the 2020 targets were met [1]. 

In 2017, UNAIDS estimated Tanzania’s progress towards the 90 – 90 – 90 targets at 70 – 62 – 63, while the Tanzania HIV Impact Survey (THIS) reported 61 – 94 – 87 [3]. These figures imply high levels of coverage for treatment with some progress still needed in testing. In particular, there are at least 30% of PLHIV in Tanzania who are yet to know their status and have not accessed testing. It also shows that at least 6% and 13% of PLHIV with a known diagnosis are either not linked to care or not virally suppressed, respectively. Health system characteristics such as lack of coordination between the multiple data sources likely contribute to the variability observed between these two estimates [4]. Additionally, a linear progression through the HIV care cascade assumed by the targets over-simplifies the actual pathways clients navigate as they engage the health system to access services [5]. 

Evidence from studies conducted in Tanzania reveals demand-side (patient-related) barriers to HIV testing, including differences in care-seeking behavior between sexes and risk groups including adolescents, low perception of HIV risk, fear of a positive HIV test result, and denial of HIV test result [6,7,8,9,10,11,12]. Others include socio-cultural issues such as stigma, contextual issues such as illiteracy, and food shortage [13,14]. Supply-side barriers include structural issues related to the availability of health services, distance to facility, antiretroviral stock-out, the severe shortage of human resources for health, poor quality of service delivery, decreasing donor funds, and policy-related issues [13,15,16,17,18]. 

Understanding the barriers PLHIV faces [14,19] and how these influence their care-seeking behavior as they navigate the HIV treatment cascade will help support the development of effective strategies to boost their engagement and retention in each specific setting. Our research aimed to investigate the alignment between HIV-related care-seeking behavior in rural Tanzania with HIV care and treatment service availability to inform interventions and ultimately ameliorate access. We also set out to identify and categorize supply- and demand-side barriers facing PLHIV living in rural Tanzania as they navigate through the HIV treatment cascade to access care.

## 2. Materials and Methods

Our study setting was Shinyanga, a largely rural region located in the northwestern zone of Tanzania. Spanning a land area of nearly 19,000 sq. km, the population density is estimated at 81 persons per sq. km. Poverty and illiteracy are widespread, and the main occupation is farming [20]. In 2019, an estimated 67,000 PLHIV were residing in the region [21], and HIV services were available to various degrees across most health facilities. HIV testing is available at most dispensaries and during community outreach activities, while treatment can be accessed in clinics and hospitals. Less than 10% of the population has health insurance coverage, however, HIV services are provided free of charge at the point of care at both public and not-for-profit private health facilities.

Employing patient-pathway analysis (PPA) methods described by Hanson et al. [22,23], we explored existing HIV data sources to derive estimates of HIV service access. PPA methods simulate a patient’s journey as s(he) navigates the health system to access services. Though mostly described within the context of tuberculosis (TB) programs, the method is adaptable to HIV or other disease programs.

Data sources used in our study reflect the latest data available and include the 2016–2017 THIS (see Table 1), the Tanzanian Health Facility Registry (HFR), the National AIDS Control Program (NACP) HIV Data Handbook, the 2014 – 2015 Tanzanian Service Provision Assessment (TSPA), and the District Health Information System version 2 (DHIS2) summary report. We charted the number of PLHIV accessing care at each step of the treatment cascade with the number of HIV services available. Only data reported for the Shinyanga region were used for computations. The comprehensive list of health facilities in the Shinyanga region in 2019 was obtained from the HFR, which is an online registry of all health facilities in Tanzania [24]. Besides administrative information about the facilities, the types of services provided are included in the HFR, which was extracted for each facility. 

Health facilities were categorized into health system levels by adopting the TSPA convention and aligned with the standardized naming convention proposed by Hanson et al. [22,26]. There were four health system levels: Level 1 to 4 (Table 2). Level 1 was assigned to all household/community-based health services (e.g., health outreaches, campaigns, chemist shops). To allow for more granularity of information, we deviated from the initial TSPA convention by designating Level 1a to dispensaries, clinics, laboratories, and health centers at the village and ward levels and Level 1b to district-level hospitals. Hospitals at the regional, zonal, and national levels were assigned Levels 2, 3, and 4, respectively, though there were no level 3 and 4 hospitals in the Shinyanga region. Facilities designated as ‘closed’ or ‘under construction’ in the HFR were excluded. Facilities were also split into two main sectors: public and private sectors. The public sector included all government-owned facilities, while the private sector included for-profit and not-for-profit facilities. The sector “community” was assigned where services were provided out-of-facility. 

### 2.1. HIV Testing Access

We estimated HIV testing access by multiplying the proportion of THIS respondents with HIV tests done per sector with the proportion of facilities with HIV testing available. This shows the proportion of respondents likely to access facilities with HIV testing capacity during their last clinic visit.

$$HIV\;\;access\;=\%\;THIS\;respondents\;with\;HIV\;test\;done\;\times \;\%\;facilities\;with\;HIV\;testing\;capactiy\;\left(by\;sector\right)$$

The proportion of respondents with HIV tests done per sector and type of facility was estimated using the place of last HIV test, reported as “HIV testing location” in the THIS data. To obtain the most recent estimates, only data for adult respondents who had an HIV test during the last 12 months of the survey (July 2016 to June 2017) were included in the analysis. There were 12 options to select from for “HIV testing location” in the THIS and no clear demarcation of whether facilities visited belonged to the public or private sectors. Therefore, we estimated the proportion of respondents per sector based on the HIV testing capacity coverage reported per sector in the HFR and the DHIS2 summary report, at the level of respondents’ HIV testing location. All ‘HIV testing locations’ in the THIS outside health facilities were counted as ‘community sector’ and level 1. HIV testing capacity was defined as the proportion of facilities across sectors and levels with HIV testing services available (i.e., having rapid diagnostic tests (RDTs) in addition to the number of community-based HIV testing activities). Facility level data from the HFR was triangulated with the DHIS2 summary report to ascertain HIV test availability. 

### 2.2. CD4 and HIV Viral Load (HVL) Testing Access 

CD4 and HVL testing availability estimate the percentage of health facilities per sector and level with CD4 count and HVL testing capacity. To estimate this, data in the DHIS2 summary report was primarily used. It was compared with relevant facility data extracted from the HFR, and where a difference existed, the DHIS2 report data was used. CD4 testing was conducted within facilities if the requisite equipment was available, otherwise, samples were transported for processing to nearby facilities. For HVL testing, all facilities in the Shinyanga region transported HVL samples to Bugando Hospital, Mwanza (Zonal Hospital) for processing, a distance of between 170–200 km. The coverage data represent facilities with a direct sample transportation arrangement in place with Bugando Hospital.

$$CD4\;or\;HVL\;\;access\;=\%\;THIS\;respondents\;with\;HIV\;test\;done\;\times \;\%\;facilities\;with\;CD4\;or\;HVL\;testing\;capactiy\;\left(by\;sector\right)$$

### 2.3. Treatment Access and Outcome 

Treatment access is an estimate of the proportion of respondents who had their last HIV test done in facilities where antiretroviral therapy (ART) services were also available. It represents the likelihood of ART initiation at the same facilities during the last HIV testing if a positive HIV diagnosis was obtained, by sector and level. The availability of ART services in health facilities in the Shinyanga region by sector and level was estimated as the proportion of facilities where ART services were available in 2019. The numbers used in the PPA were estimated similarly to the CD4/HVL testing capacity data (i.e., from the DHIS2 summary report compared with data from the HFR). 

Treatment outcome was defined based on the proportion of patients who accessed treatment services in the Shinyanga region who were virally suppressed as per all HVL tests done for the second quarter in 2019. It was obtained from the report of the Shinyanga region in the NACP HIV data handbook. Treatment outcome data were not available by sector or level.

$$Treatment\;\;access\;=\%\;THIS\;respondents\;with\;HIV\;test\;done\;\times \;\%\;facilities\;with\;ART\;capactiy\;\left(by\;sector\right)$$

**Figure 1:**
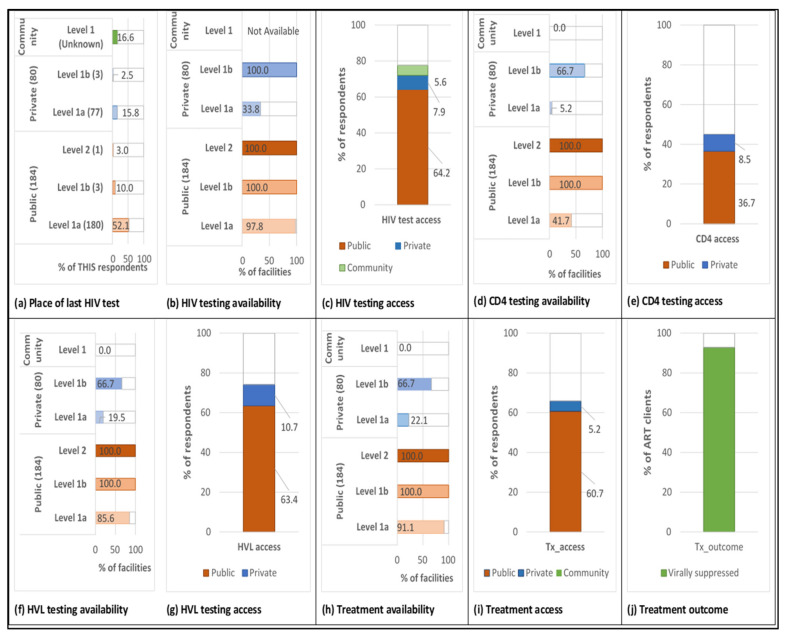
Patient-pathway analysis visual. (**a**) Number of facilities and place of last HIV test: Estimated number of facilities in each sector and proportion of respondents who had an HIV test within the last 1 year in the 2016–2017 Tanzania HIV Impact Survey (THIS); (**b**) HIV testing availability: % of facilities or community-based programmes with HIV testing services available obtained from Tanzania Health Facility Registry (HFR) and DHIS2 summary report; (**c**) HIV testing access: % of THIS respondents who tested for HIV in the public and private sector, by level; Testing availability (**d**) CD4 count and (**f**) HIV viral load): % of facilities with HIV diagnostic services (CD4 count and HIV viral load) available as obtained from DHIS2 summary report; Testing access (**e**) CD4 count and (**g**) HIV viral load): % of PLHIV who could access HIV diagnostic services at the same facility as their last HIV test by sector and level; (**h**) Treatment availability: % of facilities where ARV are available as obtained from the Tanzania HFR and DHIS2 summary report; (**i**) Treatment access: % of PLHIV who could access ART services at the same facility as their last HIV test (by sector and level); (**j**) Treatment outcome: % of PLHIV who are virally suppressed out of all viral tests done in the region for the second quarter of 2019 from the National AIDS Control Programme HIV data handbook.

For ease of comprehension, all the estimated PPA components described above were visualized using Microsoft Excel (2016) Microsoft Corporation, Redmond, USA (Figure 1).

### 2.4. Ethical Considerations

The PPA methodology utilizes only aggregated, publicly available data and does not involve primary data collection. It is a secondary data analysis and is therefore exempt from ethics review (or separate informed consent). The appropriate data transfer agreement required to use the existing aggregated data from the HIV data handbook and the DHIS 2 summary report was in place with the National AIDS Control Program. Data from the health facility registry Tanzania is freely available online from the official website [24]. THIS data is available upon direct request from the Population-based HIV Impact Assessment (PHIA) project website [27].

## 3. Results

According to the Tanzania Health Facility Registry, in 2019, there were 264 functional health facilities in the Shinyanga region. The frequency of facilities by level is shown in Figure 1a. Most facilities were level 1a (68%), one was level 2 (i.e., the Shinyanga Regional Hospital) (3%), while the rest were level 1b, reflecting the rural aspect of the region. In terms of ownership, two-thirds of facilities were government-owned (i.e., public sector), 8% were owned by private-not-for-profit (mostly faith-based) organizations, and the rest were private-for-profit. Community-based services existed for HIV-related care as well as other prevalent diseases and were supported by both private and public sector facilities and other non-governmental organizations (NGOs).

### 3.1. HIV Testing Access

Most respondents (65%) in THIS had their last HIV test done in public health facilities (Figure 1a). The rest were equally shared between the private sector and community services. Most tests were done at the level 1a facilities (68%). Most community HIV testing services were either health campaigns or mobile Voluntary Counseling and Testing. HIV testing services were available in almost all public health facilities except for a small proportion of level 1a facilities, less than 3% (Figure 1b). In the private sector, all level 1b facilities provided HIV testing, but HIV testing was provided by only 34% of level 1a facilities. The non-availability of community sector testing availability reports is likely explained by the fact that community outreaches and campaigns are usually led by facilities and other organizations in the public and private alike, and the data is usually poorly delineated. Region-wide, 78% of clients accessed HIV testing services at locations with HIV testing capacity during their last HIV test (Figure 1c). The public sector represented over 80% of those locations, while the private sector amounted to only 10%. HIV testing access through community-based services accounted for about 6% of all clients, which was still less than facility-based tests.

### 3.2. CD4 and HIV Viral Load Testing Access

Figure 1d,f shows that in the public sector, CD4 testing was available at all level 1b and 2 facilities, while in the private sector, it was available mainly in level 1b facilities. CD4 testing capacity is generally limited in level 1a facilities, especially in the private sector. For HVL testing availability, the figures show that all level 1b and 2 public and 66.7% of level 1b private facilities had a direct sample transport system with Bugando, where HVL testing platforms are available. Most level 1a facilities (85.6%) in the public sector also had direct HVL testing arrangements with Bugando, compared with only one-fifth of similar facilities in the private sector. CD4 and HVL testing are primarily facility-based, therefore no share of coverage was observed at the community level. Compared to facilities where HIV treatment was available, HVL testing was also available at all level 1b and 2 facilities but only in 94% and 88% of level 1a facilities in the public and private sectors, respectively. The proportion of those who had their last HIV test done at a facility with CD4 count testing capacity was 36.6% and 8.5% in the public and private sector, respectively (Figure 1e). For HVL testing, the proportion of those who had their last HIV test done at a facility with HVL testing access in the case of a positive HIV result was 63.4% and 10.7% in the public and private sector, respectively (Figure 1g).

### 3.3. Treatment Access and Outcome

Figure 1h shows that ART was available in almost all public health facilities except for about 9% of level 1a facilities. The private sector, however, shows that only two-thirds of level 1b and 22.1% of level 1a facilities could provide ART services. The proportion of respondents who could access ART services at the same facility where they had their last HIV test was 60.7% and 5.2% in the public and private sector, respectively (Figure 1i). As of the second quarter in 2019, the viral suppression rate estimated among 6410 PLHIV on ART with HVL test results in the Shinyanga region was 91.5% (Figure 1j) [21].

## 4. Discussion

Our study investigated the alignment of HIV care-seeking behavior with HIV service availability in the Shinyanga region, Tanzania. Public health facilities offering HIV services were more available than private facilities, and the majority (>64%) of people sought HIV testing in these facilities. Compared to CD4 count testing, access to HVL testing was considerably higher (>74% vs. 45%), reflecting the changes in guidance. Similarly, ART access and consequently HVL suppression were reasonably high in the public sector.

The wider availability of publicly owned health facilities in the Shinyanga region aligned with the distribution pattern prevalent in Tanzania and similar African settings, where regions with large urban centers attracted more private sector investment in health [24,28,29]. The low-income status prevalent in this region likely restricted existing private facilities to level 1. The regulated status of HIV services provided to clients by the government (through donor funds) free at the point of service in public and private not-for-profit facilities also characterizes many HIV programs in Africa [30]. This likely explains why the majority of PLHIV accessed care in public facilities [31,32].

The considerably high HIV testing access in the Shinyanga region was likely linked to the availability of free HIV testing in dispensaries in addition to the involvement of non-formally laboratory-trained personnel in HIV testing. Free HIV services are a common feature of HIV treatment programs in sub-Saharan Africa (SSA) and contribute to the increased HIV testing coverage observed [33]. Innovative testing models, such as HIV self-testing, which target specific populations not sufficiently engaged in the public health services (e.g., men, adolescents, and other key and vulnerable populations in Tanzania) [9,34,35] also show promise for potentially improving efficiencies and optimizing HIV testing access. The achievement of the first 90 [36,37,38,39] will, however, benefit from more engagement of private sector level 1a facilities through the provision of training or other incentives, such as the provision of self-testing or ART refill. The effective coordination of community HIV-testing strategies in Uganda showed an increasing number of PLHIV tested [40]. This finding agrees with our hypothesis, which suggests that optimal private sector involvement and effective community HIV-testing coordination in Shinyanga could increase testing access by 10% and 12%, respectively.

In Shinyanga, CD4 count and HVL testing were only available from level 1b (district-level) facilities and upwards. At lower levels, whether in the public or private sector, capacity for CD4 count testing, as well as other HIV-related diagnostic tests, existed where the facility was supported by an implementing partner or other external funders, a situation which raises concern about sustainability [41,42]. The test and treat strategy, the recommended and increasing donor support for routine HVL testing, and the consequent decrease in CD4 count testing as per guidelines all likely contributed to the lower access to CD4 count testing observed in our study [43,44,45]. A declining trend of CD4 count measurement has been observed in many southern African countries since the beginning of the Test and Treat era in alignment with our findings [46,47,48]. Despite this, the considerably high access to HVL testing observed in Shinyanga likely reflects the donor-funded status of this service. A well-coordinated laboratory network involving the local ministry of health, implementing partners, donors, and private sector stakeholders in resource-limited settings have been proposed to optimize diagnostic services [49,50]. Additionally, the new guidelines recommend exploring differentiated service delivery to expand HVL testing access in the private sector, which may also benefit the early identification of treatment failure and minimize drug resistance [51].

Evidence from other African settings shows considerable patronage, even among PLHIV who access free treatment, of the informal health system, including faith healers and traditional medicine practitioners, which are poorly documented [52,53,54]. The consequences of these practices included medical pluralism, poor adherence to ART, drug resistance, and ultimately the withdrawal of therapy. Attaining global ART treatment targets in Shinyanga will involve capturing those lost in the continuum of formal HIV care and accommodating the needs of others who prefer and can afford the additional cost of patronizing the private sector. A promising example involved exploring accredited pharmacies (e.g., Duka La Dawa Baridi (DLDB)) for making community HIV services increasingly available in rural Tanzania [55]. Coordinated collaboration between these various practitioners involving the development of an efficient referral system or incentivizing care linkage, especially for practitioners in the formal and informal private sector, appears to be a viable option.

The high viral suppression seen in Shinyanga signifies considerably successful treatment outcomes among those on ART and aligns with the evidence showing high viral load suppression in rural East Africa when treatment is well managed [56,57]. Ultimately, managing the HIV response in Shinyanga should focus on closing the testing and treatment access gaps.

In Tanzania, both supply- and demand-side barriers to access have been documented. Supply-side barriers included poor staff attitude towards patients during service delivery [58,59]. Discriminatory attitudes towards PLHIV have also been documented [60]. Affordability did not seem to constitute a major barrier in Tanzania since HIV services are mostly provided free at the point of delivery. Depending on where services were accessed, however, PLHIV in Shinyanga might still have to pay out of pocket for other medications or laboratory services not catered for in the free program. The distribution of health facilities and the kinds of services available varied widely across regions. Rural dwellers usually had more limited access to basic health services due to fewer health centers over a wide geographical area and the consequent long distances to access health services [61]. A follow-up to this is the lack of basic amenities, such as essential medicines, equipment, adequate staff, and laboratory services in most dispensaries and health centers representing the health facilities most widely spread in the rural areas [62,63,64,65]. Limited availability of services has also been revealed in terms of inflexible work hours [58,62].

From a demand-side perspective, the perception of poor attitudes during treatment by staff, long waiting times, unclear clinic procedures, and insufficient confidentiality of interaction in health facilities all constitute barriers [11,66]. Being asymptomatic, belief in alternative medicine and lack of disclosure are other barriers at the individual level. The fear of unintentional disclosure of status due to frequent clinic visits and perceived stigma, whether self-imposed or otherwise, all discourage PLHIV from actively engaging in the health system [10,67,68]. Though HIV treatment is provided free of charge, patients incur costs as they need to travel far distances to health facilities to access the free care. This is a commonly documented barrier, especially in rural Africa [11,14,69,70]. Widespread illiteracy levels, as well as a lack of information about HIV or awareness about services, represent other barriers that impede uptake even where services are available [65]. Ehrenkranz et al. have proposed a cyclical cascade to discern more realistic pathways that clients navigate and articulate the attending barriers [5].

Our study had several limitations mainly related to the assumptions made from the use of secondary data. The THIS survey that we used to estimate the locations where respondents accessed their last HIV test did not delineate the facility locations by sector (i.e., whether public or private). We assumed a distribution among respondents similar to the distribution of these facilities in the Shinyanga HFR for respondents accessing facility types that could either have been public or private. Besides the faith-based clinics and hospitals, and a few NGO-funded clinics, health services in the Shinyanga region are predominantly provided by the public sector. It may be that the figures were underestimated for the private sector. Similarly, the hospital-type locations were not demarcated as either district or regional. We assumed a proportional distribution (i.e., that the proportion of clients who had their last HIV test at a hospital followed a similar pattern as the distribution of level 1b and 2 hospitals in the region). It may be that the proportion of respondents attributed to the level 1b hospital (regional hospital) was underestimated, but this bias does not impact the overall proportion of clients accessing the HIV services reported. Additionally, the PPA estimates for accessing diagnostic and treatment services at the location of the last HIV test were based on the coverage of these services. Several factors have, however, been documented regarding why those testing positive may not necessarily access these services even where they exist [11,71]. Therefore, actual access may have been underestimated. Our estimates are likely conservative, considering that most of those testing positive are willing to access services, especially as they are free.

## 5. Conclusions

In Shinyanga, most people were likely to have access to HIV testing when they visited a public health facility. Access to CD4 count and HVL testing was more likely for PLHIV who had had their HIV tests done in public compared to private health facilities. Future HIV program implementation may benefit from research exploring differentiated service delivery to expand access to diagnostic services in the private sector and may improve CD4 and HVL testing. Similarly, engaging accredited pharmacies and private clinics to make HIV services increasingly available within the community, especially in rural areas, may improve access to treatment. Ultimately, to reach the last mile toward ending the HIV epidemic, the main focus in the short term should be testing and synergies with the private sector, as there remains a proportion of the population who may only be reached if the private sector is engaged or innovative options like self-testing are implemented.

## Figures and Tables

**Table 1:** Primary data sources for the PPA.

Component	Sub-Component	Data Source(s)
Number of facilities	Formal private and public facilities	Tanzania Health Facility registry (HFR) [24]
Place of last HIV test	HIV testing availability	HFR [24]
		District Health Information Software (DHIS2) summary report [25]
		2014 – 2015 Tanzanian Service Provision Assessment Survey (TSPA) [26]
	HIV testing access	2016 – 2017 Tanzanian HIV impact survey (THIS) [3]
HIV diagnostics at the place of last HIV test	*CD4 count and HIV viral load test availability	HFR [24];
		DHIS2 summary report
	CD4 count and HIV viral load access	THIS [3]
HIV treatment at the place of last HIV test	HIV treatment availability	THIS [3]
		DHIS2 summary report
	HIV treatment access	THIS [24]
		HFR [24]
HIV treatment outcome	HIV treatment outcome (viral suppression)	National AIDS Control Program (NACP) HIV data handbook [21]

* CD4—cluster of differentiation 4, a glycoprotein co-receptor for the T-cell receptor

**Table 2:** Health facility categorization.

Data Source	Facility Type	Facility Sector	Level
Tanzania Health Facility Registry (HFR)	Clinic	Public and Private	1a
	Clinic – Dental Clinic	Private	1a
	Clinic – Diagnostic Centre	Private	1a
	Clinic – Dialysis Clinic	Private	1a
	Clinic – Eye Clinic	Private	1a
	Clinic – General Clinic	Private	1a
	Clinic – Medical Clinic	Public and Private	1a
	Clinic – Optometry Clinic	Private	1a
	Clinic – Other Clinic	Public and Private	1a
	Clinic – Physiotherapy Clinic	Private	1a
	Clinic – Polyclinic	Private	1a
	Clinic – Specialized clinic/Polyclinic; Super specialized clinic/ Polyclinic	Private	1a
	Dispensary	Public and Private	1a
	Health Center	Public and Private	1a
	Health Labs	Public and Private	1a
	Health Labs – Level IA1 (Health Center Laboratory)	Private	1a
	Health Labs – Level IA2 (Dispensary Laboratory)	Public and Private	1a
	Health Labs – Level III Multipurpose Health Laboratory	Private	1a
	Health Labs – Level III single purpose Health Laboratory	Private	1a
	Health Labs – Specimen collection point	Private	1a
	Hospital	Private	1a
	Hospital – Council Designated Hospital	Public and Private	1a
	Hospital – Other Hospital	Public and Private	1a
	Maternity and Nursing Home	Private	1a
	Maternity Home	Public and Private	1a
	Nursing Home	Public and Private	1
	Health Labs – Level IIA2 (District Laboratory)	Private	1b
	Hospital – District Hospital	Public	1b
	Hospital – Hospital at District Level	Public and Private	1b
	Hospital – Hospital at Regional Level	Private	2
	Hospital – Referral Hospital at Regional Level	Private	2
	Hospital – Regional Referral Hospital	Public	2
	Hospital – Hospital at Zonal Level	Public and Private	3
	Hospital – Referral Hospital at Zonal Level	Public and Private	3
	Hospital – Super Specialized Hospital at National Level	Public and Private	4
	Hospital – Referral Hospital at National Level	Public	4
2016–2017 Tanzania HIV Impact Survey (THIS)	ANC clinic	Public and Private	1a
	At home	Community	1a
	Blood donating center	Public	1b, 2
	Campaigns	Community	1a
	Health clinic/Facility	Public and Private	1a
	Hospital inpatient wards	Public and Private	1b, 2
	Hospital outpatient clinic	Public and Private	1b, 2
	Mobile VCT	Community	1a
	Social events	Community	1a
	STI clinic	Public and Private	1a
	TB clinic	Public and Private	1a
	VCT facility	Public and Private	1a

Legend: ANC – Antenatal clinic; VCT – Voluntary Counselling and Testing; STI – Sexually Transmitted Infection; TB – Tuberculosis; IA1, IA2, and IIA2 – codes assigned to represent laboratory level in the HFR.

## Data Availability

Restrictions apply to the availability of the 2016 – 2017 THIS adult dataset used in our study. Data were obtained from the PHIA project and are available upon direct request to the PHIA project (https://phia.icap.columbia.edu/ (accessed on 23 December 2020)). Summary DHIS2 data report was obtained through the Data Transfer Agreement between the Amsterdam Institute for Global Health and Development and the National AIDS Control Programme (NACP) authorized by the National Institute for Medical Research (NIMR) reference number NIMR/HQ/R.8a/Vol.IX/ 2711, dated 2 September 2019. The health facility registry data used in the study is publicly available at the HFR site (http://hfrportal.moh.go.tz/ (accessed on 7 May 2020)) and the sources are included in the manuscript.

## Abbreviations

ART—Antiretroviral therapy; CD4—Cluster of differentiation 4; DHIS2—District Health Information System version 2; HFR—Tanzania Health Facility Registry; HIV—Human Immunodeficiency virus; HVL—HIV viral load; LMIC—Low and Middle Income countries; NACP—National AIDS Control Program; NGO—Non-governmental organization; PHIA—Population-based HIV Impact Assessment; PLHIV—People living with HIV; PPA—Patient pathway analysis; RDT—Rapid diagnostic tests; SSA—Sub-saharan Africa; TB—Tuberculosis; THIS—Tanzania HIV Impact Survey; TSPA—Tanzania Service Provision Assessment; UNAIDS—Joint United Nations Program on HIV/AIDS.
